# Time-Restricted Eating and Metabolic Syndrome: Current Status and Future Perspectives

**DOI:** 10.3390/nu13010221

**Published:** 2021-01-14

**Authors:** Iwona Świątkiewicz, Alina Woźniak, Pam R. Taub

**Affiliations:** 1 Department of Cardiology and Internal Medicine, Collegium Medicum, Nicolaus Copernicus University, 85-094 Bydgoszcz, Poland; 2 Division of Cardiovascular Medicine, University of California San Diego, La Jolla, CA 92037, USA; ptaub@health.ucsd.edu; 3 Department of Medical Biology and Biochemistry, Collegium Medicum, Nicolaus Copernicus University, 85-092 Bydgoszcz, Poland; alina-wozniak@wp.pl

**Keywords:** time-restricted eating, metabolic syndrome, abdominal obesity, hyperglycemia, dyslipidemia, inflammation, oxidative stress, cardiometabolic risk, circadian rhythm, eating pattern

## Abstract

Metabolic syndrome (MetS) occurs in ~30% of adults and is associated with increased risk of cardiovascular disease and diabetes mellitus. MetS reflects the clustering of individual cardiometabolic risk factors including central obesity, elevated fasting plasma glucose, dyslipidemia, and elevated blood pressure. Erratic eating patterns such as eating over a prolonged period per day and irregular meal timing are common in patients with MetS. Misalignment between daily rhythms of food intake and circadian timing system can contribute to circadian rhythm disruption which results in abnormal metabolic regulation and adversely impacts cardiometabolic health. Novel approaches which aim at restoring robust circadian rhythms through modification of timing and duration of daily eating represent a promising strategy for patients with MetS. Restricting eating period during a day (time-restricted eating, TRE) can aid in mitigating circadian disruption and improving cardiometabolic outcomes. Previous pilot TRE study of patients with MetS showed the feasibility of TRE and improvements in body weight and fat, abdominal obesity, atherogenic lipids, and blood pressure, which were observed despite no overt attempt to change diet quantity and quality or physical activity. The present article aims at giving an overview of TRE human studies of individuals with MetS or its components, summarizing current clinical evidence for improving cardiometabolic health through TRE intervention in these populations, and presenting future perspectives for an implementation of TRE to treat and prevent MetS. Previous TRE trials laid the groundwork and indicate a need for further clinical research including large-scale controlled trials to determine TRE efficacy for reducing long-term cardiometabolic risk, providing tools for sustained lifestyle changes and, ultimately, improving overall health in individuals with MetS.

## 1. Introduction

Metabolic syndrome (MetS) occurs in approximately 30% of adults and is associated with increased cardiometabolic morbidity and mortality [1,2,3,4]. The presence of MetS doubles the long-term risk of developing cardiovascular disease (CVD) and cardiovascular mortality, and is associated with a 5-fold increase in the risk of type 2 diabetes mellitus (T2DM) [2,3]. The MetS is characterized by multiple risk factors for CVD and T2DM including central obesity, elevated fasting plasma glucose (FPG), dyslipidemia, and elevated blood pressure (BP) [3,5]. In general, MetS reflects the clustering of individual cardiometabolic risk factors related to abdominal obesity and insulin resistance [2]. Once CVD or T2DM develops, the number of MetS components contributes to disease progression and patient outcome [3].

Erratic eating patterns such as eating over a prolonged period per day and eating more than three meals a day are common [6,7,8,9,10,11] and are associated with obesity, T2DM, MetS, and CVD [12,13,14,15]. It has been shown that over 50% of people eat during a period >15 h every day, only ~10% of adults habitually maintain fasting of ≥12 h per day [7,9,10]. In an American population of patients with MetS, the eating window, defined as the time interval during a day in which 95% of all calorie-containing ingestion events occur, was ~15 h [16]. It has been also demonstrated that irregular meal timing adversely impacts cardiometabolic health [14]. Misalignment between daily rhythms of food intake and circadian timing system can contribute to circadian rhythm disruption which results in abnormal metabolic regulation, disruption of metabolic homeostasis, and increased cardiometabolic risks [17,18,19,20,21].

Obesity, physical inactivity, and erratic dietary patterns are among the most important modifiable risk factors that contribute to the pathogenesis of MetS and its cardiometabolic outcomes [2,22,23]. Improving the diet and structured lifestyle interventions are currently the first line of treatment for MetS, and are critical to prevent disease progression [6,22,23,24,25]. However, due to poor patient adherence the effectiveness of interventions that address disturbed metabolic homeostasis through changes in quality and quantity of nutrition, increase in physical activity, and promotion of weight loss with low-calorie diets is low [25,26,27]. Moreover, these strategies are difficult to sustain long-term, so their efficacy at improving cardiometabolic risks in patients with MetS is limited.

Novel approaches which aim at mitigating circadian disruption through modifying timing and duration of daily food intake represent a promising strategy for patients with MetS [18,24,28,29,30,31,32]. Time-restricted eating (TRE) is a lifestyle intervention in which eating is restricted to a reduced, fixed number of hours per day, which supports an adequate fastening period [10,12,18,30,33]. The underlying hypothesis for the effectiveness of TRE in metabolic disorders is that imposing eating–fasting cycles will restore robust circadian rhythms and improve metabolic regulatory mechanisms, which can favorably impact cardiometabolic outcomes. Recently, several small-scale clinical studies employing TRE were performed in individuals with metabolic disorders [10,11,16,34,35,36,37,38,39,40,41,42,43,44]. A few recent reviews evaluated the metabolic effects of TRE in both animal and human studies; however, they were not focused exclusively on the populations with MetS [18,45,46,47].

The present article aims at giving an overview of the protocols and results of TRE clinical human studies of individuals with MetS or its components, summarizing current clinical evidence for improving cardiometabolic outcomes and overall health through TRE intervention in these populations, and presenting future perspectives for an implementation of TRE to prevent and treat MetS.

## 2. Diagnostic Criteria of MetS and Its Components

According to the International Diabetes Federation Joint Interim Statement criteria, MetS is defined in the presence of ≥3 of the following 5 risk factors: increased waist circumference (population- and country-specific definitions should be used), elevated FPG (≥100 mg/dL or drug treatment of elevated glucose), elevated BP (systolic ≥130 and/or diastolic ≥85 mm Hg or antihypertensive drug treatment for diagnosed hypertension), hypertriglyceridemia (≥150 mg/dL or drug treatment for this lipid abnormality), and reduced high-density lipoprotein cholesterol (HDL-C) (<40 mg/dL in males and <50 mg/dL in females or drug treatment for this lipid abnormality) [3,48]. The cut-off values of increased waist circumference for males are 94 cm, 102 cm, and 90 cm in European, US, and other populations including the Asian population, respectively [3,48]. For females, these values are 80 cm, 88 cm, and 80 cm, respectively. Most patients with T2DM will have the MetS by the proposed criteria [3,48].

Overweight and obesity, typically defined as the body mass index (BMI) of ≥25 kg/m^2^ and ≥30 kg/m^2^, respectively, significantly increase the probability of metabolic disorders including MetS and are major risk factors for CVD and T2DM [1,2,3,49,50]. For the BMI, the optimal cut-off point for the identification of metabolic disorders in the European population is 27.2 kg/m^2^ [51]. The waist circumference measurement is recommended for those with BMI of 25 to 34.9 kg/m^2^ to provide additional information on CVD risk; however, if BMI is >30 kg/m^2^, central obesity can be assumed and waist circumference does not need to be measured [3,48]. However, owing to the need for screening of individuals with a metabolically obese normal weight, the measuring of waist circumference should be considered when BMI is ≥22.5 kg/m^2^ in females and ≥23.8 kg/m^2^ in males [50,51,52].

In the adult US population, the prevalence of overweight and obesity was reported as high as 71% and 40%, respectively [2]. Overweight or obesity occur in ~83%, ~76%, and ~74% of subjects with T2DM, hypertension, and dyslipidemia, respectively [53]. Importantly, MetS was diagnosed in ~33% of overweight and ~65% of obese individuals [50]. The prevalence of elevated waist circumference indicating abdominal obesity, hypertriglyceridemia, elevated BP, and hyperglycemia in the adult US population were reported as ~56%, ~24%, ~24%, and ~20%, respectively [54]. In the Metabolic syndrome and Arteries REsearch (MARE) Consortium of 34,821 subjects with MetS, mostly from the European countries, two clusters of factors were observed in 12% and 13% of subjects; first, hypertriglyceridemia, elevated BP and increased waist circumference, and second, elevated FPG, elevated BP and increased waist circumference [1].

## 3. Impact of Circadian Rhythms and Circadian Rhythm Disruption on Metabolic Homeostasis

The central pathophysiological feature of MetS is a disruption in metabolic homeostasis with excess central adiposity followed by insulin resistance, impaired FPG and/or impaired glucose tolerance [5,55]. Other metabolic and neuroendocrine disorders such as reduced level of adiponectin, leptin resistance, aberrant ghrelin and resistin levels, and a deficiency of melatonin were also linked to obesity-related disorders including MetS [56,57,58,59,60,61,62]. In addition, low-grade inflammation and oxidative stress contribute to the pathophysiology of obesity, atherosclerosis, T2DM, and MetS [5,63,64,65,66]. Elevated C-reactive protein (CRP) levels, which are related to insulin resistance and MetS, are independent predictors of CVD events [63,67,68,69,70,71]. Oxidative stress is associated with adiposity, impaired insulin signaling pathways, insulin resistance, and increased risk of cardiometabolic diseases [64,72,73,74].

Circadian rhythms are a regulatory mechanism that maintains metabolic homeostasis and coordinates the processes of nutrition, physical activity, and sleep such that anabolic and catabolic pathways are active at different times of the 24 h day [18,33,75,76,77,78,79]. Systematic analyses of circadian gene expression in the mouse liver revealed a dominant role of eating pattern on daily rhythms in gene expression [76]. A daily rhythm in the eating–fasting cycle supports a robust rhythm in mRNAs and proteins, which regulate metabolic processes such as gluconeogenesis, glycolysis, protein synthesis, lipid synthesis and oxidation, and mitochondrial function [18,33,76,78,80,81]. For example, insulin sensitivity, β cell responsiveness, the thermic effect of food, and fatty acids oxidation are all higher in humans in the morning than later during the day, which suggests that metabolism is optimized for food intake in the morning [18,20,47,78,82]. It has been demonstrated in human studies that eating in alignment with circadian rhythms, e.g., by increasing food intake at breakfast time and reducing it at dinnertime, can result in weight loss and improvements in glycemic control and lipid levels [81,83]. Metabolic homeostasis is also significantly influenced by circadian-related hormones such as cortisol, melatonin, adipokines, resistin, and ghrelin [56,57,58,59,60,61,62]. For example, melatonin regulates energy flow by influencing the intensity and circadian distribution of metabolic processes including the proper synthesis, secretion and action of insulin and, consequently, glucose homeostasis [18,56,57,60,78].

Misalignment between circadian timing system and daily rhythms of food intake or sleep–wake behavior as a result of genetic, environmental or behavioral factors can contribute to circadian rhythm disruption which adversely impacts metabolic homeostasis and cardiovascular function [17,21,78,84,85,86,87]. Circadian disruption results in abnormal constant activation or suppression of metabolic regulatory mechanisms, which can cause abnormal glucose metabolism with defective glucose tolerance and insulin resistance [17,18,19,20]. Circadian disruption was also shown to be associated with an increase of oxidative stress, activation of inflammation, as well as disordered regulation and aberrant secretion of circadian-related hormones [17,59,60,73]. A large body of epidemiologic and clinical evidence indicated that circadian disruption due to shift work, sleep deprivation, and erratic eating patterns is associated with an increased risk of MetS and its components, T2DM, and CVD [13,77,82,88,89,90,91,92]. For example, late-night caloric intake increased the risk of coronary heart disease by as much as 55%, even after controlling for diet and lifestyle [13]. Therefore, lifestyle interventions that improve daily rhythms of behavior including eating pattern can favorably impact circadian rhythms and are expected to improve cardiometabolic health [12,29,81].

## 4. Effects of TRE in Animals and Healthy Humans

A number of animal-based studies indicated that maintaining an appropriate daily rhythm of eating-fasting cycles sustains robust circadian rhythms, which improves cellular metabolism [80,93,94,95,96,97]. TRE is a circadian rhythm-reinforcing lifestyle which introduces a consistent period of 12–16 h fasting every day, which in various pathways mitigates circadian disruption and promotes improved metabolic homeostasis [12,18,76]. TRE restores normal levels and/or normal daily rhythms in several mRNAs, proteins, and metabolites that are implicated in metabolic homeostasis of glucose, lipids, redox, and mitochondria function, and regulates circulating adiponectin and leptin levels. TRE was shown to exert pleiotropic beneficial effects on multiple organ systems of mice (liver, muscle, white adipose tissue, brown adipose tissue, gut, and brain) [93,94,95,96] and insects (muscle, heart, brain) [97]. TRE had beneficial impact on mitochondrial structure and function which translates to improved physiology in animals, such as improved endurance and cardiac contractility [93,94,97]. In animal models, an implementation of TRE for 8–12 h per day prevented fatty liver, dyslipidemia, and glucose intolerance, as well as resulted in improvements in glycemic control, insulin levels, inflammation, body weight regulation, energy expenditure, motor coordination, cardiac contractility, sleep, and endurance level [12,18,80,93,94,95,96,97]. Beneficial effects of TRE were dose-dependent with better effects post 9 h protocol compared to 12 or 15 h [47,94]. TRE reversed many aspects of MetS by decreasing body weight, adiposity, glucose intolerance, as well as cholesterol and triglycerides (TG) levels, and improving heart function. Importantly, TRE resulted in cardiometabolic benefits even when food intake and/or body weight was matched to the control group [93,95]. Thus, TRE can impart benefits irrespective of nutrition quantity and quality and seems to be both preventive and therapeutic for cardiometabolic diseases [18]. Therefore, there is a potential to adopt TRE for improving cardiometabolic health in humans with metabolic disorders including MetS [18,28,30].

Several small-scale TRE human studies were conducted in healthy individuals with normal weight, both young including subjects undergoing resistance training or recreationally active [98,99,100,101,102] and in middle aged and older adults [103,104,105]. These studies demonstrated that TRE is a feasible and well tolerated dietary strategy across the lifespan. Various beneficial effects of TRE, which were initially shown in animal studies, were demonstrated in healthy humans. TRE resulted in a decrease in energy intake [98,101], body weight even without reducing energy intake [98,99], body fat [99,100,102,104], BP [99], blood glucose and TG [100], glucose tolerance [105], leptin and inflammatory markers [100], and hunger [105], as well as an increase in adiponectin and HDL-C [99,100] and muscular strength and endurance capacity [101,102,105]. In healthy non-obese adults, TRE did not impact lean mass, muscular performance, bone density, and nutrient intake [100,101,102,105]. However, in several studies of healthy subjects, no significant impact of TRE was found on body weight [100,101], body fat [101], glucose and insulin levels [99,102], lipids [99,100,102], inflammatory markers [105], and cortisol pattern [99,102]. Moreover, healthy adults with normal weight not aligning TRE to the circadian rhythm (i.e., concentrating food intake to late afternoon or evening) exhibited worsened cardiometabolic outcomes such as elevated FPG and morning glucose intolerance [103]. Several review articles addressed TRE effects in healthy humans and indicated that this dietary strategy can be beneficial for subjects with cardiometabolic diseases including MetS [12,18,28,30,33,46,47,76].

## 5. Characterization of TRE Trials in Humans with MetS or Its Components

Several small-scale TRE human studies were performed in individuals with metabolic disorders such as MetS or its components [10,11,16,34,35,36,37,38,39,40,41,42,43,44]. The protocols of these studies differed in terms of objectives, study design, inclusion criteria, population, sample size, duration of TRE eating window and intervention, diet prescription, and methods of recording food intake. In addition to the objectives associated with a feasibility of TRE intervention, TRE studies aimed at evaluating changes in cardiometabolic biomarkers. The main characteristics of TRE trials which were conducted in humans with MetS or its components are shown in Table 1.

### 5.1. Study Design

TRE human studies of populations with metabolic disorders were designed either as randomized controlled trials [11,34,35,42,43,44] or, more frequently, single-arm studies including one group of participants with pre-post intervention design [10,16,35,37,38,39,40,41]. A lack of control group in the single-arm studies is a potential limitation implying that the results of these studies should be interpreted with caution. Several studies were pilot trials which aimed primarily at testing a feasibility of TRE intervention [10,11,34,38,41].

Most TRE studies were based on small sample size, usually less than 20 participants in single-arm studies (except for the study of Kesztyüs et al. [41] which involved 40 subjects). On average, 30 participants were included in randomized controlled trials with 6–20 participants in each arm. The study of Lowe et al. [44] included 116 participants but only 50 of them participated in an in-person cohort (with 25 participants in each arm) which was subject to cardiometabolic outcomes analyses.

### 5.2. Inclusion Criteria and Participants

Populations with metabolic disorders included young [10,39,40,43], midlife [11,16,34,36,37,41,42,44] and older subjects [38]. The inclusion criteria comprised an increased BMI indicating overweight or obesity [10,11,34,35,36,38,39,40,42,43,44], diagnosed MetS or ≥1 of its components such as increased waist circumference [16,37,41], and the diagnosis of prediabetes or T2DM or high risk of developing T2DM [35,37,41]. To qualify as having prediabetes in the study of Sutton et al. [35], participants needed to exhibit both elevated glycated hemoglobin (HbA1c) and impaired glucose tolerance based on an oral glucose tolerance test. In the study of Hutchinson et al. [37], the high risk of T2DM was determined based on the Australian Type 2 Diabetes Risk Assessment Tool (AUSDRISK) score including the risk factors such as age, sex, ethnicity, parental history of diabetes, history of high blood glucose, use of antihypertensive medications, physical inactivity, and obesity defined as an increased BMI and/or waist circumference.

To our knowledge, only the study of Wilkinson et al. [16] enrolled exclusively patients with diagnosed MetS. In that study, eligible participants had to satisfy ≥3 of MetS criteria and self-reported dietary intake of ≥14 h per day, regular daytime schedule of activity, and self-reported habitual sleep duration of >6.5 h. The exclusion criteria included diagnosis of diabetes, shift work, history of major adverse cardiac events, active medical conditions, history of eating disorder or bariatric surgery, participation in the weight-management program, special diet for other reasons, substance abuse, depression, sleep apnea, and treatment with antidepressants, medication affecting glucose metabolism or appetite, or immunosuppression. Patients in that study had central obesity and elevated TG, FPG and HbA1c. The majority of patients (84%) received pharmacotherapy for elevated BP and dyslipidemia such as antihypertensive medications in 79% and statins in 63% of patients.

Several TRE human studies involved individuals with the presence of one or more components of MetS [11,35,36,37,38,42,43,44]. The typical multifactorial clusters of MetS components were as follows: increased waist circumference, elevated FPG and elevated BP [37,38] as well as BMI >30 kg/m^2^ (indicating central obesity) accompanied with elevated FPG [35], elevated BP [11,42] or decreased HDL-C [36]. Participants in other TRE studies had either BMI >30 kg/m^2^ indicating central obesity [10,39,40,43,44] or increased waist circumference [41]. Importantly, most patients (63%) with abdominal obesity in the study of Kesztyüs et al. [41] received antihypertensive medications but only one patient was treated by statin. In addition, a few TRE studies of subjects with metabolic disorders comprised erratic eating pattern (mostly defined as habitually eating over a period ≥14 h per day) as an inclusion criterion [10,11,16].

### 5.3. TRE Window and Duration of Intervention

The protocols of published TRE studies defined an eating window, i.e., a number of hours per day when food can be taken, to vary from as long as 12 h [10,35,39,40] to as short as 4 h [42] with the mean of 8–10 h in the majority of trials [11,34,36,38,41,43,44]. As a result, TRE intervention introduced a consistent period of fasting every day which could vary from 12 to 20 h. The effects of TRE interventions with different eating windows (e.g., 4 vs. 6 h and 6 vs. 12 h) were evaluated through direct comparisons between subgroups with different eating windows [42] or the use of a crossover study design [35,39,40,43]. In several studies, the start and end of the TRE eating window in the day was self-selected by participants [10,11,16,38,41]. In most studies, however, the start and end time of the eating window was imposed by the study protocol [35,36,37,39,40,42,43,44]. Also, the effects of early TRE, i.e., eating early in the day [10,35,36,39,40] and delayed TRE, i.e., with a phase delay to late hours in the day [37,44] were evaluated.

The designs of TRE studies included a different length of intervention period varying from short-term intervention of 4 days [39,40] to long-term intervention of 16 weeks with the follow-up period up to 1 year [10]. The most frequent duration of TRE intervention was in the range of 8–12 weeks [11,16,34,36,41,42,44].

### 5.4. TRE Intervention

During TRE intervention, study participants were asked to restrict their food intake daily to a number of hours a day which was defined as an eating window and to fast for the remaining hours. They were also instructed to start the TRE intervention by selecting an eating window that best suits their lifestyle based on his/her baseline eating pattern or the start and end time of eating during a day were defined in the study protocols. For example, in the study of Wilkinson et al. [16] including patients with MetS, the 10-h TRE eating window had to be between 7 a.m. and 9 p.m., with the last meal (including non-water beverage) consumed at least 2 h prior to the typical bedtime. In this study, the 10-h interval was entered in a smartphone application, so participants could visualize their chosen daily eating window and consume all meals within this interval.

In several TRE studies of individuals with metabolic disorders, TRE was the only intervention, and participants were not instructed to change their habits regarding the quality, quantity, or caloric content of their diet; however, a meeting with a dietitian in-person for behavioral nutritional counseling was included in some protocols [10,16,34,38,41]. In some studies, special diet prescriptions were implemented, such as meals were provided with matched nutrients [35,39,40,43], or ad libitum nutrition was recommended but within TRE eating window [10,11,16,34,36,44]. Specifically, in the trial of Sutton et al. [35], the obese and prediabetic male participants undergoing TRE intervention were required to eat only food provided by study staff, were fed enough food to maintain their weight, and ate all meals while being monitored by study staff. This protocol allowed testing of whether TRE has cardiometabolic benefits in the absence of weight loss.

In addition, in most TRE studies, participants with metabolic disorders were not instructed to change their habits regarding physical activity during TRE intervention [10,16,34,35,38,39,40,41]. However, in some studies, physical activity control was conducted using actigraphy devices or mobile phone applications [11,16,36,37,43,44].

### 5.5. Use of the MyCircadianClock Application

The myCircadianClock application (mCC app) for a smartphone is the validated HIPPA (Health Insurance Portability and Accountability Act) compliant method that was developed at the Salk Institute for Biological Studies (La Jolla, CA, USA) for recording food intake and monitoring adherence to TRE intervention in real time [10,11,16]. The server side of the app is designed to run multiple independent clinical studies with individual customization allowing study-specific customization by the investigator and user-specific customizations by participants. The mCC app serves as an electronic food, activity, and sleep diary [10]. In studies which used mCC app, participants were asked to record all food intake, physical activity, and sleep quantity/quality every day during TRE intervention [10,11,16]. Data from the app can be used to assess participant adherence, eating window, calorie intake, physical activity, and sleep. The adherence to mCC app use is high, mainly due to the simplicity of logging food metadata by making photo of food item or entering the name of food from the list.

### 5.6. Calorie Intake Assessment

In TRE studies using the mCC app, the food intake data (photo and/or annotation entries) were downloaded from the server-side of the app and dietary analyses were performed by a registered dietitian to calculate the overall calorie intake and estimate calories from macronutrient component, usually by using the Caloric King database [11,16]. Also, dietary records were used by TRE studies with ad libitum intake to document energy intake [36] or no food records were applied [44].

It is known that participants with obesity underreport energy intake by 20–40% when using food diaries [108] and self-reporting of adherence by patients is generally not accurate [109]. Implementing mobile apps to assess caloric intake in real-time seems to be more accurate than assessing it by self-report food records. It was shown that the false-negative rate (i.e., food consumed but not logged) using the mCC app is ~10% [10]. Controlling unlogged dietary events is challenging, and the ideal control which is expensive and difficult in humans would involve direct observation of all dietary events [35]. However, such a strategy is unlikely to provide sustainable approach for long-term TRE intervention.

### 5.7. Use of Continuous Glucose Monitor

In several TRE studies, a continuous glucose monitor (CGM) was used for an evaluation of glucose metabolism [11,16,37,39,40,43]. CGM measures interstitial fluid glucose every 15 min, using a subcutaneous sensor. CGM estimates blood glucose levels with high accuracy that correlates with those obtained from either venous or capillary blood. By using CGM, mean daily glucose level, post-prandial glucose response, mean amplitude of glycemic excursion, continuous overall net glycemic action, and the glycemic variability index can be calculated. CGM can also be used to track adherence to TRE intervention.

### 5.8. Physical Activity and Sleep Assessment

Actigraphy devices such as actiwatches or pedometers were used in several TRE studies to assess a level of physical activity and the duration, quality, and timing of sleep [10,11,16,36,37,38,43,44]. Sleep was also analyzed by mCC app and questionnaires such as the Pittsburgh Sleep Quality Index questionnaire [16,36].

### 5.9. Body Composition Analysis

The body composition measurements were usually performed using bioelectrical impedance technology or, less frequently, dual-energy X-ray absorptiometry (DXA) that is more accurate [11,16,36,44]. Bioelectrical impedance-measured percentage body fat and visceral fat, and greater muscle mass percentage, or DXA-measured fat mass, lean mass, or visceral fat were evaluated.

### 5.10. Primary Outcomes

The primary outcomes of TRE human clinical studies of individuals with metabolic disorders are depicted in Table 1. In general, they included the measures associated with an adherence and safety of TRE intervention, as well as post-TRE changes in cardiometabolic outcomes such as body weight, fat mass percentage, FPG, mean daily glucose levels obtained by CGM, glucose tolerance, postprandial insulin, insulin sensitivity, circadian blood profiles of glucose and insulin, and energy expenditure.

## 6. Major Findings of TRE Trials in Humans with MetS or Its Components

Major findings of TRE trials in humans with MetS or its components are displayed in Table 2.

### 6.1. Adherence to TRE Intervention

Based on mCC app logging, surveys through the custom mobile study app, or food diaries records, it has been demonstrated that the adherence to TRE intervention is high [11,16,36,41,44]. The adherence to TRE, defined as the proportion of days in which the TRE eating window was achieved during the whole TRE intervention period, was shown to be as high as ~83–86% [11,16,36,41,44] and 98% of participants were adherent to required meal times when following early TRE while under rigorous control [35]. In addition, ~63% of patients with MetS were still to some degree adherent to TRE at ~16 months after completing the intervention despite they were not advised to continue TRE beyond the intervention period [16]. Self-selecting of TRE eating window seems to facilitate maintaining an adherence to TRE intervention long term [16].

### 6.2. Eating Pattern

Eating window duration significantly declined (on average by one-third) during TRE intervention and participants were able to maintain a shortened daily eating window for an extended period of time during TRE interventions lasting 12–16 weeks [10,11,16,36,41] and even up to 16 months [10,16]. In the pilot study of an American population with MetS, TRE resulted in shortening daily eating window from ~15 h to ~10 h with nightly fasting of ~14 h during the 12-week period [16]. In addition, that eating pattern was achieved by delaying and advancing in meal timing rather than skipping meals and regularity in timing of meals was increased in that study. Also, in the study of Chow et al. [11] of obese adults, allowing unrestricted intake within an 8-h TRE resulted in a decrease in the number of eating occasions of ~20%.

### 6.3. Body Weight, Waist Circumference, and Body Composition

Several TRE studies of individuals with metabolic disorders focused on changes in body weight [10,11,36,42,44]. Typically, 8–16 weeks of TRE with various eating windows of 4–10 h resulted in weight loss by ~3–4% in overweight or obese subjects both young [10] and older [11,36,38,42], prediabetic or diabetic subjects with abdominal obesity [37,41], and patients with diagnosed MetS [16]. Importantly, 4-h and 6-h TRE produced comparable reduction in body weight by ~3% in obese adults compared to controls [42]. Also, a reduction in BMI by ~3% was observed post-TRE in patients with MetS [16]. The post-TRE weight reduction of 3% in patients with MetS is comparable to the effects of calorie restriction combined with exercise in studies of subjects with glucose intolerance [110,111]. However, no significant changes in BP or lipids were reported in these studies in contrast to the results of Wilkinson et al. [16]. In the study of Lowe et al. [44], a significant decrease in weight was observed in the TRE group albeit to smaller extent than in other TRE studies. However, the change was not significant compared to the control group. It should be noted that most participants in that study self-reported their weight changes using a Bluetooth weighing scale that was linked to a custom app. In addition, no tools such as mCC app were used to monitor adherence. These factors could be a limitation of the study results. No change of body weight was observed after 10 weeks of 8 h TRE in overweight individuals despite a decrease in adiposity [34]. In the study of Sutton et al. [35], a lack of reduction in body weight post-TRE intervention in obese and prediabetic subjects was intentional and resulted from the special diet prescription which aimed at maintaining weight.

A decrease in body fat (percentage and mass) by 3–4% was observed post TRE in obese participants who exhibited weight loss [11,41,42], in patients with MetS independently of change in weight [16], and in overweight subjects without weight loss [34]. In addition, a reduction in visceral fat (up to ~11% of DXA-measured fat mass in obese midlife subjects) and a decrease in waist circumference (~3–4%) were also observed post TRE in patients with MetS and abdominal obesity [11,16,41]. A decrease in waist circumference in patients with MetS correlated with change in body weight and eating window [16]. Moreover, greater restriction of the eating window was associated with greater loss of DXA-measured fat mass and visceral fat [11]. However, in a few other TRE studies of obese midlife sedentary subjects and obese individuals with high risk of T2DM, no change in body fat was found despite a decrease in body weight [36,37].

A decrease in body weight may account for some beneficial effects of TRE on cardiometabolic outcomes. However, in the study of patients with MetS, eating interval or weight did not account for all changes in cardiometabolic health [16]. In the study of Sutton et al. [35], post-TRE metabolic benefits were observed in the absence of weight loss.

Although lean mass was maintained post-TRE in healthy subjects with normal weight including those undergoing regular resistance training or recreational activity [100,101,102,105], a significant loss of lean mass was observed in obese midlife subjects [11,44]. It would be of interest to address the question as to whether TRE leads to preferential loss of lean (versus fat) mass and whether this effect is offset by changes in protein content or timing of protein consumption [44]. Also, it would be important to explore a potential risk of post-TRE muscle loss in elderly populations, especially that no measurements of body composition were made in the TRE study of overweight/obese individuals of age ≥65 years, in which weight loss was observed [38].

### 6.4. Calorie Intake

A decrease in calorie intake by ~9–20% was reported in TRE studies of overweight or obese individuals including patients with MetS despite no recommendations to change the diet quantity and quality during the intervention [10,11,16,34,36,42]. In obese adults without overt metabolic disease, energy intake was reduced by as much as 20% without calorie counting, partly due to a decrease in consumption of alcoholic beverages and late-night snacks [10,36]. Importantly, it was observed that 4-h and 6-h TRE produced comparable decrease in energy intake (by ~550 kcal/day) without calorie counting in obese adults compared to controls [42]. Importantly, diet quality did not change post-TRE in that study.

A decrease in energy intake may account for some beneficial effects of TRE on body weight and metabolic outcomes. However, in the study of Sutton et al. [35], which was a strictly controlled crossover feeding trial, post-TRE metabolic benefits were observed in the absence of calorie restriction and weight loss.

In some TRE studies, unintentional reduction in energy intake concurrent with a truncated TRE eating window was observed, which suggests that implementation of TRE without caloric reduction is difficult to achieve in humans due to spontaneous energy restriction during the study period [16,36].

### 6.5. Glucose Metabolism

A decrease in mean glucose obtained from CGM was reported post-TRE in obese but otherwise healthy young adults [39,40] and obese subjects with high risk of T2DM [37], as well as FPG and fasting glucose obtained by CGM in overweight and obese midlife adults [11,34]. However, the reported changes in fasting glucose obtained by CGM were not significant when compared to non-TRE group in the study of Chow et al. [11]. TRE improved glucose tolerance in obese men with prediabetes with or without weight loss [35,37]. Both early and delayed TRE produced similar improvements in glucose tolerance; however, only early TRE significantly reduced fasting glucose obtained by CGM in obese men with high risk of T2DM [37]. Sutton et al. [35] showed that limiting food intake prior to 3 p.m. (early TRE) is beneficial for glycemic control in obese and prediabetic men, independent of weight change and reducing caloric intake. In that study, early TRE improved insulin sensitivity, insulin resistance, β-cell function, and decreased postprandial insulin, however, did not result in a change in FPG. Importantly, the reductions in insulin levels were the largest in participants with worse baseline hyperinsulinemia and persisted long term after completing TRE intervention. In addition, insulin and insulin resistance were improved in a few other TRE studies of overweight or obese subjects [34,36,42]. The 4 h and 6 h TRE produced similar reductions in insulin resistance in obese individuals [42]. When baseline FPG and HbA1c were elevated, TRE also resulted in improvements of glycemic control such as lowering HbA1c by ~4% in subjects with abdominal obesity and patients with MetS [16,41]. In addition, TRE improved nocturnal glycemic control in men with overweight or obesity [43]. It has been postulated that prolonged fasting during TRE intervention may improve glycemic control by the metabolic switch, which occurs when changing from fed to fasted state [30]. The metabolic switch induces hepatocyte production of ketone bodies, increasing insulin sensitivity and decreasing fat accumulation.

However, some TRE studies of individuals with metabolic disorders showed that some glycemic measures remain unchanged [11,35,36,38,42,43,44]. No significant improvements in average glucose obtained by CGM, FPG, and insulin resistance were observed in patients with MetS [16]. Importantly, however, moderate changes in the desirable direction of FPG, fasting insulin and HbA1c were reported post-TRE in patients with MetS [16]. This suggests that patients with more impaired glucose metabolism may benefit more from TRE compared to lower-risk individuals.

### 6.6. Lipid Metabolism

In the pilot study of patients with MetS, TRE led to significant reduction of atherogenic lipids such as total cholesterol (~7%), low-density lipoprotein cholesterol (LDL-C) (~11%) and non-high-density lipoprotein cholesterol levels (~9%), and a favorable trend towards reduction was found for TG and LDL-C particle number [16]. These changes in cholesterol levels cannot be explained solely by weight loss as a decrease of 5% of body weight was shown to result in a 3–5% reduction in LDL-C, which is lower than the post-TRE level [112]. In the study of Wilkinson et al. [16], a 3% weight loss was accompanied by 11% reduction in LDL-C. A decrease in TG was also observed post-TRE in obese subjects including prediabetic men [11,37]. In the study of Chow et al. [11], however, the changes were not significant when compared to the non-TRE group.

However, the observed effects of TRE on plasma lipids were highly variable and usually unchanged in other studies of overweight or obese subjects, including individuals with abdominal obesity, T2DM, and prediabetes [11,35,36,37,41,42,44]. Importantly, early TRE resulted in an increase in fasting TG which was likely due to a long fasting period and TG re-esterification following lipolysis [35,39,40]. It should be noted, however, that the benefits of TRE are minimal in patients with normal lipid levels and the benefits are more pronounced in those with elevated baseline lipids [35,36].

### 6.7. Neuroendocrine, Metabolic, Oxidative Stress, and Inflammation Biomarkers

An impact of TRE on metabolic and neuroendocrine hormones in humans remains poorly investigated [39,40,98]. Early 6-h TRE resulted in a decrease in morning and mean ghrelin levels and altered diurnal patterns in cortisol [39,40].

Scant data about the impact of TRE on oxidative stress suggest a reduction of lipid peroxidation (lowering of 8-isoprostane levels by 14%) in obese subjects including men with prediabetes, which may indicate that TRE potentially reduces a risk of atherosclerosis [35,42]. Importantly, both 4-h and 6-h TRE produced comparable reduction in 8-isoprostane levels (up to 37%) in obese adults [42]. The decrease in oxidative stress may be related to improvements in insulin resistance. It has been demonstrated that insulin signaling is impaired during oxidative stress, resulting in insulin resistance of the cell [72,73]. Also, insulin sensitivity improves when administering antioxidants such as vitamin E [113].

Data on the effects of TRE on the inflammatory markers in humans are limited and inconsistent [16,35,41,42,100]. No significant improvement in high-sensitivity CRP level was detected in the pilot study of patients with MetS [16]. Also, TRE had no effect on circulating CRP, tumor necrosis factor-α, and interleukin-6 in obese subjects including men with prediabetes [35,42]. Altered expression of several circadian clock genes was reported post TRE in overweight and obese individuals [39,43]. There is some evidence that TRE may have a beneficial effect on the gut microbiome [18,114].

### 6.8. Blood Pressure

TRE resulted in a reduction of systolic (up to ~5–11 mmHg) and diastolic (up to ~7–10 mmHg) BP in a few studies of middle-aged obese subjects [36,44], patients with MetS [16] and obese adults with prediabetes, even in the absence of weight loss [35]. In patients with MetS, systolic and diastolic BP decreased by ~4% and 8%, respectively, which is comparable or even greater to that expected by weight loss through other means [16,115]. Large reductions in BP in the study of early TRE, which were comparable with the antihypertensive effects of angiotensin-converting enzyme inhibitors, may suggest that the improvements in BP were driven by the decrease in insulin levels and increased natriuresis by shifting salt intake to earlier in the daytime [35,116,117]. In addition, TRE can increase the efficacy of pharmacotherapy that also exhibits circadian rhythms [118]. However, no change in BP was observed in a few other studies of obese adults, so further evidence of the impact of TRE on BP is needed [42,44].

### 6.9. Behavioral Effects and Safety

Multiple beneficial behavioral outcomes of TRE were observed in various populations including patients with MetS, such as an improvement in self-reported sleep, energy level, subjective feeling of hunger at bedtime, quality of life and well-being [10,16,35,37,38,39,40,43,107]. Early 6-h TRE resulted in a decrease in appetite, and, despite the longer daily fasting duration, feeling of hunger in the evening, which may facilitate weight loss [35,40]. In the study of Ravussin et al. [40], the early TRE facilitated weight loss primarily by decreasing appetite rather than by increasing energy expenditure. Aligning food intake with circadian rhythms may, therefore, be a good strategy for reducing appetite and losing weight. Physical activity usually remained unchanged in TRE studies if participants were not instructed to change their habits regarding physical activity [11,16]. Work schedules, family commitments and social events have been considered as barriers to long-term implementation of TRE [43,47].

Self-reported adverse events (such as nausea, vomiting, headaches, dizziness, increased thirst, constipation, and diarrhea) were rare during TRE intervention [35,41] or no events were reported during 8–10-h TRE [16,42,106]. Importantly, TRE was safe even when short eating windows of 4 or 6 h were implemented [42]. The findings of previous TRE studies indicated that mild adverse effects may occur at the onset of TRE, but they usually disappear when the participant becomes adjusted to the diet [41,42].

## 7. Effectiveness of TRE in Humans with MetS or Its Components: Summary of Clinical Evidence

**Figure 1:**
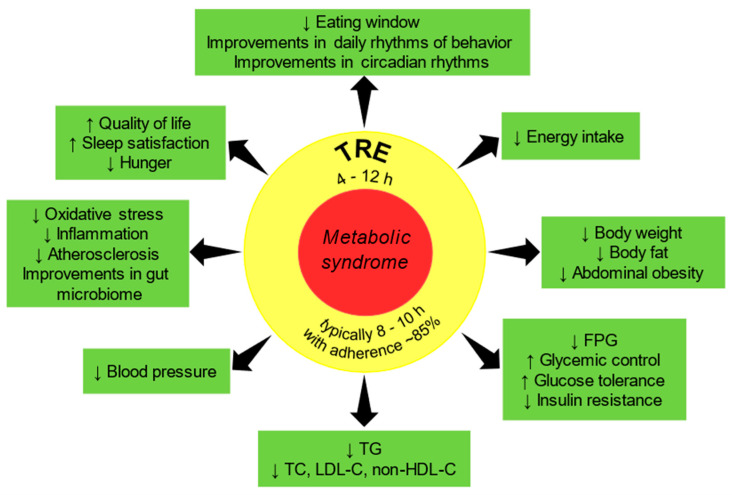
Demonstrated and potential cardiometabolic benefits of time-restricted eating (TRE). FPG: fasting plasma glucose; LDL-C: low-density lipoprotein cholesterol; non-HDL-C: non-high-density lipoprotein cholesterol; TC: total cholesterol: TG: triglycerides. The arrow pointing down indicates a decrease and the arrow pointing up indicates an increase.

Patients with MetS have abnormal regulation of glucose and fat metabolism, increased inflammation and oxidative stress, and disrupted regulation of metabolic hormones. These changes lead to widespread chronic diseases from obesity, T2DM, to CVD. Previous TRE human studies provided the evidence that adopting a simple TRE pattern intervention is feasible and can lead to restoring rhythmic daily behavior resulting in a reduction of cardiometabolic risks and improvements of health in populations with metabolic disorders including patients with MetS (Figure 1).

Specifically, several small-scale studies in humans with metabolic disorders including one pilot study of patients with MetS showed that TRE resulted in weight loss and a decrease in eating window duration, fat mass, and energy intake, as well as improved glucose tolerance, insulin resistance, glycemic control, lipids, self-reported sleep, and reduced BP [10,11,16,35,36,37,38,39,40,41,42,43,107]. In the pilot study of an American population of patients with MetS, the improvements in body weight and fat, waist circumference, atherogenic lipids, and BP were observed despite no overt attempt to change diet quantity and quality or physical activity, and cardiometabolic benefits were mostly independent of change in body weight and eating window [16].

In the populations with central obesity, elevated BP and elevated FPG, TRE resulted in a decrease in weight, fasting glucose levels obtained by CGM, fasting TG, feeling of hunger, as well as improvements in glucose tolerance and quality of life [37,38]. In the populations with central obesity and elevated BP, TRE improved body weight and fat, fasting glucose levels obtained by CGM, insulin resistance, and oxidative stress [11,42]. Prediabetic and obese men who followed TRE intervention without losing weight exhibited improvements in insulin sensitivity, BP, oxidative stress, and reduced appetite [35]. TRE has been readily adoptable in the populations with central obesity and resulted in reducing body weight, waist circumference, feeling of hunger, ghrelin, systolic BP, and improvements in insulin resistance, glycemic control, oxidative stress, sleep, and well-being [10,36,39,40,41,43].

Obese adults with a prolonged daily eating window undergoing TRE intervention easily improved body weight and fat without calorie counting [10,11,16]. Moreover, TRE studies of subjects with metabolic disorders, mainly involving US populations, reported high patient adherence to TRE intervention which supports sustainability of this therapeutic approach, especially if it is monitored by using the mCC app [10,11,16,35,36,38,44].

Based on the current evidence provided by TRE studies of patients with metabolic disease, the American Heart Association published a scientific statement which supports the notion that maintaining a consistent eating period during the day and prolonged overnight fasting can mitigate cardiometabolic risks and prevent CVD [119].

## 8. Knowledge Gaps and Future Perspectives for TRE in MetS

TRE offers a promising strategy for the prevention and treatment of cardiometabolic disease including MetS. However, most TRE studies were focused on evaluating the feasibility and/or effects of TRE intervention in healthy individuals with normal weight and overweight or obese adults without overt metabolic disease. The in-depth interpretation and generalization of the effectiveness of TRE in MetS is hampered by several factors such as a limited number of TRE human studies, various populations, small sample size, short-term duration of TRE intervention and follow-up, various TRE eating windows implemented at different times during a day, and study design without non-TRE control group. While only one pilot TRE study (19 patients) provides results that are specific to an American population with diagnosed MetS [16], the findings of several other TRE studies that included individuals with one or more components of MetS suggest potential usefulness of TRE for improving the outcome of subjects with cardiometabolic disease.

Despite the encouraging results, various aspects associated with feasibility and effectiveness of TRE intervention, and sustainability of weight loss and cardiometabolic benefits long term in patients with MetS require further research. First, we recall that the enrollment criterion to target a population with diagnosed MetS has been uncommon in previous TRE studies. Also, a few TRE studies, including the pilot study of subjects with MetS, involved participants with a prolonged eating window (≥14 h) [10,11,16]. Therefore, the findings of these studies may not be applicable to subjects with metabolic disease and shorter eating window. It appears that further clinical research including exclusively patients with diagnosed MetS regardless of baseline eating window is needed. Moreover, data on the feasibility and effectiveness of TRE in European populations with MetS or its components are presently very much limited [41].

Second, large-scale randomized controlled trials with longer duration of TRE intervention and long-term follow-up are desirable. Specifically, TRE trials with long intervention period (>12 months) would be useful to test if a decrease in energy intake without calorie counting followed by weight loss, which was observed previously during shorter TRE interventions, persists and is sustainable long term. In addition, most previous TRE studies of individuals with metabolic disorders included midlife subjects while only a few trials involved young and older individuals [38,39,40]. Given a risk of age-related muscle loss, more evidence of TRE effects on nutrient intake, muscle strength and bone mineral density, especially in elderly populations, is desirable [46,103,120]. Modulating the gut microbiota through TRE to reverse microbial dysbiosis associated with circadian misalignment may mitigate metabolic risk [18,114], so the effects of TRE on the gut microbiome with potential benefit in MetS warrant further investigation.

Third, the effects of TRE on glucose and lipid metabolism were found to be highly variable in TRE studies of various populations with metabolic disorders. Specifically, the results of potentially unfavorable TRE effects on lipids require further investigation [35,39,40]. Also, the effects of TRE on metabolic and neuroendocrine homeostasis have been poorly investigated. Data on the effects of TRE on inflammatory markers in humans, including MetS patients, are limited and inconsistent. There are also only scant data about the impact of TRE on oxidative stress. Therefore, extending the protocols used in TRE studies by including more comprehensive range of biomarkers is needed.

Fourth, the optimal TRE eating duration and timing require special attention in further research. Beneficial effects of TRE were observed with various TRE protocols; however, it is likely that greater eating period restriction would produce more benefits [11,47]. On the other hand, while short eating windows could result in worsening of adherence and nutrient intake, extending the eating window beyond 12 h seems to produce no beneficial effects [98]. Available data seem to support a 10 h TRE eating window as this window still produces benefit and long-term adherence is better [10,16]. Also, more data to address the contribution of TRE timing (early vs. late eating) to the observed metabolic effects are needed. Although delayed TRE is likely more attractive and more amenable to long-term adherence, it might not be optimal for the metabolic advantages of TRE.

Finally, the mechanisms of beneficial effects of TRE in humans are still poorly understood. Importantly, measurement of circadian rhythms has not been performed in previous TRE human studies although it would be useful for understanding the mechanisms of TRE effects [17]. Extensions of study protocols with circadian rhythms measurements, mitochondrial function evaluation, and additional tools for recording food intake and chrono-nutrition assessment are desirable. Also, the roles of weight loss, energy restriction, decreasing appetite, delimitation of an eating window, changes in energy expenditure and caloric balance in beneficial metabolic effects of TRE require elucidation. Available data suggest that the benefits of TRE go beyond the benefits of caloric restriction and weight loss [16,35].

The rationale for the extant TRE clinical human studies builds largely on extensive mechanistic molecular and physiological studies of circadian rhythms, eating patterns, and metabolic homeostasis, both in animal models and human studies. Although TRE is promising for prevention and treatment of cardiometabolic diseases, evidence of the effectiveness of TRE in MetS is limited, thus necessitating focused human studies. The extensions of study protocols are important to advance an understanding of the roles of metabolic regulatory mechanisms and circadian system in the pathophysiology of MetS and its complications. An important example of the paucity of information relates to the implementation and cardiometabolic effects of TRE as an alternative approach in the management of the European populations with MetS. Some new results are expected in the near future as indicated by the search of clinical trials registries, which shows that a few TRE studies in the European and American populations of patients with metabolic disorders including MetS and shift workers were recently undertaken [121]. The acquisition of TRE-related data for different populations and the use of consistent protocols would facilitate creation of joint databases and comparative analysis of patients with MetS from different countries and continents.

The TRE intervention represents an approach that can have translational impacts leading to improvements in health and a reduction of risks for cardiometabolic diseases, disability, and premature death. Projected short-term improvements include beneficial changes in body weight and composition, sleep and physical activity, performance and wellbeing. The findings of previous studies suggest that TRE is achievable and can help adults with obesity or other metabolic disorders to reduce calorie intake and lose weight without calorie counting, and improve cardiometabolic health. Future TRE studies are needed and would contribute to TRE research which can ultimately lead to the development of a circadian lifestyle management plan for patients with MetS, including dietary guidelines and nutritional and health policies towards alleviating health risks.

## 9. Conclusions

Sustaining a consistent daily rhythm of eating and fasting through implementing TRE in patients with MetS can aid in restoring robust circadian rhythms and improve metabolic regulatory mechanisms, which can favorably impact cardiometabolic health. The available findings of TRE human trials laid the groundwork and indicate a need for further clinical research including large-scale randomized controlled trials to determine the efficacy of TRE for reducing long-term cardiometabolic risk, providing tools for sustained lifestyle changes and, ultimately, improving overall health in patients with MetS.

## Figures and Tables

**Table 1:** Main characteristics of time-restricted eating trials in humans with metabolic syndrome or its components.

Reference and Study Design	Inclusion Criteria	Participants	TRE Duration and EW	Meal Timing During TRE	Primary Outcome
Gill and Panda [10] One group (pre-post design), pilot feasibility study	Healthy adults BMI > 25 kg/m^2^ EW ≥ 14 h	*n* = 8 (5 M, 3 F) Overweight/obese EW 14.8 h Age 34.4 ± 2.9 y (M) 36.3 ± 4.3 y (F)	16 weeks EW 10–12 h	Self-selected EW	Change in body weight
Antoni et al. [34] Randomized controlled trial, pilot feasibility study	Healthy adults BMI 20–39 kg/m^2^	*n* = 13 (12 F, 1 M) Overweight/obese TRE: *n* = 7 (6 F, 1 M) Age 47 ± 3 y Control: *n* = 6 (F) Age 45 ± 4 y	10 weeks EW 8 h 37 min ± 22 min	TRE: breakfast delayed and dinner advanced by 1.5 h each Control: AL	Feasibility of TRE in reducing EW
Sutton et al. [35] Crossover	Males Age 35–70 y BMI 20–39 kg/m^2^ Prediabetes (based on HbA1c and OGTT)	*n* = 8 (M) Overweight/obese and prediabetic Age 56 ± 9 y	5 weeks in each condition EW 6 or 12 h	eTRE: 8 a.m.–2 p.m. (dinner before 3 p.m.) Control: 8 a.m.–8 p.m. 3 meals provided and matched across arms	Changes in glucose tolerance, postprandial insulin, and insulin sensitivity
Gabel et al. [36,106,107] Matched historical controls, pilot study	Age 25–65 y BMI 30–45 kg/m^2^ Sedentary to lightly active	*n* = 46 (41 F, 5 M) Obese TRE: *n* = 23 (20 F, 3 M) Age 50 ± 2 y Control: *n* = 23 (21 F, 2 M) Age 48 ± 2 y	12 weeks EW 8 h	TRE: 10 a.m.–6 p.m. Control: AL	Change in body weight
Hutchison et al. [37] Crossover	Age 30–70 y WC ≥ 102 cm High risk of T2DM	*n* = 15 (M) Obese (abdominal obesity) Age 55 ± 3 y	7 days in each condition EW 9 h	eTRE: 8 a.m.–5 p.m. dTRE: 12 p.m.–9 p.m.	Glucose response to a mixed-nutrient meal in men at risk for T2DM
Anton et al. [38] One group (pre-post design), pilot feasibility study	Age ≥ 65 y BMI 25–40 kg/m^2^ Sedentary	*n* = 10 (6 F, 4 M) Overweight/obese Age ≥ 65 y	4 weeks EW 8 h	Self-selected EW	Feasibility and safety of TRE in overweight, older adults
Jamshed et al. [39], Ravussin et al. [40] Crossover	Healthy adults Age 20–45 y BMI 25–35 kg/m^2^	*n* = 11 (4 F, 7 M) Overweight/obese Age 32 ± 7 y	4 days EW 6 or 12 h	eTRE: 8 a.m.–2 p.m. Control: 8 a.m.–8 p.m. 3 meals provided and matched across arms	Change in energy expenditure
Kesztyüs et al. [41] One group (pre-post design), pilot feasibility study	One or more components of MetS, including T2DM non requiring insulin	*n* = 40 (31 F, 9 M) Abdominal obesity Age 49.1 ± 12.4 y	12 weeks EW 8–9 h	Self-selected EW	Adherence to TRE intervention (proportion of days with fasting ≥15 h)
Wilkinson et al. [16] One group (pre-post design), pilot study	Diagnosed MetS (≥3 components) EW ≥ 14 h	*n* = 19 (6 F, 13 M) Obese with MetS EW 15.1 ± 1.1 h Age 59 ± 11.1 y	12 weeks EW 10 h	Self-selected EW	Change in mean blood glucose (CGM)
Chow et al. [11] Randomized controlled trial, feasibility study	Age 18–65 y BMI ≥ 25 kg/m^2^ EW ≥ 14 h	*n* = 20 (17 F, 3 M) Overweight/obese EW 15.2 ± 0.7 h TRE: *n* = 11 (9 F, 2 M) Age 46.5 ± 12.4 y Control: *n* = 9 (8 F, 1 M) Age 44.2 ± 12.3 y	12 weeks EW 8 h	TRE: Self-selected EW Control: AL	Change in body weight
Cienfuegos et al. [42] Randomized controlled trial	Age 18–65 y BMI 30–49.9 kg/m^2^ Sedentary or moderately active	*n* = 58 (53 F, 5 M) Obese 4-h TRE: *n* = 19 (17 F, 2 M) Age 47 ± 2 y 6-h TRE: *n* = 20 (19 F, 1 M) Age 47 ± 3 y Control: *n* = 19 (17 F, 2 M) Age 45 ± 2 y	8 weeks EW 4 or 6 h	4-h TRE: 3 p.m.–7 p.m. 6-h TRE: 1 p.m.–7 p.m. Control: AL	Change in body weight
Parr et al. [43] Randomized controlled crossover trial	Males Age 30–45 y BMI 27–35 kg/m^2^ Sedentary lifestyle	*n* = 11 (M) Overweight/obese Age 38 ± 5 y Group 1: *n* = 6 Group 2: *n* = 5	5 days EW 8 h	TRE: 10 a.m.–6 p.m. Control: 7 a.m.–10 p.m. Isoenergetic diet protocols	Circadian blood profiles of glucose and insulin
Lowe [44] Randomized controlled trial	Age 18–64 y BMI 27–43 kg/m^2^	*n* = 116 (46 F, 70 M) Overweight/obese Age 46.5 ± 10.5 y In-person TRE group: *n* = 25 (12 F, 13 M) Age 43.3 ± 11.8 y	12 weeks EW 8 h	TRE: 12 p.m.–8 p.m. CMT (consistent meal timing): 3 structured meals per day	Change in body weight

Abbreviations: AL: ad libitum; BMI: body mass index; CGM: continuous glucose monitor; dTRE: delayed-time-restricted eating; eTRE: early-time-restricted eating; EW: eating window; F: females; HbA1c: glycated hemoglobin; M: males; MetS: metabolic syndrome; OGTT: oral glucose tolerance test; T2DM: type 2 diabetes mellitus; TRE: time-restricted eating; WC: waist circumference.

**Table 2:** Major findings of time-restricted eating trials in humans with metabolic syndrome or its components.

Reference and Study Design	MetS Components	Results of TRE
Gill and Panda [10] One group (pre-post design), pilot feasibility study	Mean BMI > 30 kg/m^2^	↓ Body weight ↓ Hunger at night ↑ Morning and overall energy levels and sleep satisfaction ↓ Energy intake
Antoni et al. [34] Randomized controlled trial, pilot feasibility study	BMI 29 ± 2 kg/m^2^	↔ Body weight ↓ Body fat ↓ FPG ↔ Fasting insulin, lipids ↓ Energy intake ↓ EW by ~4.5 h
Sutton et al. [35] Crossover	BMI 32 ± 4 kg/m^2^ FPG 102 ± 9 mg/dL	↔ Body weight ↑ Insulin sensitivity, β-cell function, TG ↔ FPG, lipids, TNF-α, IL-6 ↓ BP, fasting and postprandial insulin, evening appetite, oxidative stress Adherence to TRE was 98% (rigorously controlled trial)
Gabel et al. [36,106,107] Matched historical controls, pilot study	BMI ≥ 30 kg/m^2^ HDL-C 48 ± 2 mg/dL	↓ Body weight ↔ Body fat, FPG, fasting insulin, insulin resistance, lipids, homocysteine, sleep quality/duration ↓ Energy intake ↓ Systolic BP
Hutchison et al. [37] Crossover	WC 115 ± 2 cm BP 141 ± 3/87 ± 2 mmHg FPG 105 ± 2 mg/dL	↓ Body weight ↔ Body fat, FPG ↓ Mean fasting glucose (CGM) in eTRE ↑ Glucose tolerance ↓ Fasting TG, hunger
Anton et al. [38] One group (pre-post design), pilot feasibility study	WC 109 ± 13 cm BP 146 ± 16/78 ± 12 mmHg FPG 106 ± 28 mg/dL BMI 34 ± 3 kg/m^2^	↓ Body weight ↔ Blood glucose, WC, physical and cognitive function ↑ Quality of life Adherence to TRE was 84%
Jamshed et al. [39] Ravussin et al. [40] Crossover	BMI 30 ± 3 kg/m^2^	↓ Mean 24 h glucose and glycemic excursions (CGM) ↓ Morning FPG, insulin and HOMA-IR; mean and morning ghrelin, mean appetite ↑ Morning ketones, TC, LDL-C and HDL-C; metabolic flexibility, fullness, fat oxidation ↔ Energy expenditure Altered cortisol patterns and circadian clock genes expression related to aging, stress, autophagy, oxidative stress
Kesztyüs et al. [41] One group (pre-post design), pilot feasibility study	WC 107 ± 13 cm	↓ Body weight ↓ WC ↓ HbA1c ↔ Lipids Adherence to TRE was 86%
Wilkinson et al. [16] One group (pre-post design), pilot study	Diagnosed MetS WC 109 ± 11 cm FPG 107 ± 15 mg/dL TG 161 ± 87 mg/dL HDL-C 47 ± 13 mg/dL BMI 30 ± 5 kg/m^2^	↓ Body weight ↓ WC ↓ Body fat ↔ Mean glucose (CGM), FPG, fasting insulin, HOMA-IR, HbA1c, HDL-C, TG, sleep quality ↓ TC, LDL-C, non-HDL-C ↓ Energy intake ↓ BP
Chow et al. [11] Randomized controlled trial, feasibility study	BMI 34 ± 8 kg/m^2^ BP 132 ± 13/85 ± 4 mmHg	↓ Body weight ↓ Visceral fat mass, lean mass ↓ Fasting glucose (CGM), TG ↔ Mean glucose (CGM), glucose tolerance, HbA1c, lipids No significant differences in changes of fasting glucose and TG between TRE and non-TRE groups
Cienfuegos et al. [42] Randomized controlled trial	BMI 36 ± 1 kg/m^2^ BP 135 ± 5/88 ± 2 mmHg	↓ Body weight ↓ Fat mass ↓ Energy intake ↓ Insulin resistance ↓ Oxidative stress ↔ TNF-α, IL-6
Parr et al. [43] Randomized controlled crossover trial	BMI 32 ± 2 kg/m^2^	↔ Peak and waking glucose, AUC 24 h glucose (CGM) and insulin ↓ AUC nocturnal glucose (CGM) ↑ Feeling of well-being, TG
Lowe [44] Randomized controlled trial	BMI 31 ± 5 kg/m^2^	↓ Body weight, lean mass, energy expenditure, diastolic BP ↔ Fat mass, FPG, fasting insulin, HOMA-IR, HbA1c, lipids, systolic BP No significant differences in changes of outcomes between TRE and non-TRE groups

Abbreviations: AUC: area under the curve; BMI: body mass index; BP: blood pressure; CGM: continuous glucose monitor; dTRE: delayed-time-restricted eating; eTRE: early-time-restricted eating; EW: eating window; HbA1c: glycated hemoglobin; HDL-C: high-density lipoprotein cholesterol; HOMA-IR: homeostatic model assessment for insulin resistance; IL-6: interleukin-6; LDL-C: low-density lipoprotein cholesterol; MetS: metabolic syndrome; OGTT: Oral Glucose Tolerance Test; T2DM: type 2 diabetes mellitus; TC: total cholesterol; TG: triglycerides; TNF-α: tumor necrosis factor-α; TRE: time-restricted eating; WC: waist circumference; ↑: increased levels; ↓: decreased levels; ↔: no difference.

## Data Availability

Not applicable.
